# A Fundamental Limitation of Small Diameter Single-Walled Carbon Nanotube Synthesis—A Scaling Rule of the Carbon Nanotube Yield with Catalyst Volume

**DOI:** 10.3390/ma6072633

**Published:** 2013-07-02

**Authors:** Shunsuke Sakurai, Masayasu Inaguma, Don N. Futaba, Motoo Yumura, Kenji Hata

**Affiliations:** 1 Technology Research Association for Single Wall Carbon Nanotubes (TASC), Central 5, 1-1-1 Higashi, Tsukuba, Ibaraki 305-8565, Japan; E-Mails: shunsuke-sakurai@aist.go.jp (S.S.); d-futaba@aist.go.jp (D.N.F.); m.yumura@aist.go.jp (M.Y.); 2 National Institute of Advanced Industrial Science and Technology (AIST), Central 5, 1-1-1 Higashi, Tsukuba, Ibaraki 305-8565, Japan; E-Mail: inagumam@fujikura.co.jp; 3 Japan Science and Technology Agency (JST), Honcho 4-1-8, Kawaguchi 332-0012, Japan

**Keywords:** carbon nanotube, single-walled carbon nanotube, catalyst array, synthsis

## Abstract

Understanding the fundamental mechanisms and limiting processes of the growth of single-walled carbon nanotube (SWCNT) would serve as a guide to achieve further control on structural parameters of SWCNT. In this paper, we have studied the growth kinetics of a series of SWCNT forests continuously spanning a wide range of diameters (1.9–3.2 nm), and have revealed an additional fundamental growth limiting process where the mass of the individual SWCNT is determined by the individual catalyst volume. Calculation of the conversion rate of carbon atoms into CNTs per Fe atom is 2 × 10^2^ atoms per second. This rate limiting process provides an important understanding where the larger diameter SWCNT would grow faster, and thus be more suited for mass production.

## 1. Introduction

Even after 20 years since its discovery, controlling the synthesis of carbon nanotubes (CNTs) remains the central issue for CNT research. One reason for this difficulty stems from the numerous input parameters and the numerous of structures we wish to control. For single-walled CNT (SWCNT) ensemble, there are many structural parameters which are associated to the individual CNTs, such as diameter, crystallinity, chirality, *etc.*, and another class describing how the CNTs are assembled, such as density, alignment,* etc.* Numerous researches have been carried to synthetically control these output parameters. For example, CNT diameter was controlled by tailoring the catalyst size through changing the loading level of Fe atoms in a ferritin protein [1]. Recently, reducing the diameter of SWNT in forest to the range smaller than 2 nm has been achieved in several reports by changing the relative Co-Mo content in the bimetallic catalyst [2], the addition of acetonitrile to an ethanol carbon feedstock during synthesis [3], and decoupling the catalyst formation and SWCNT growth processes [4]. The importance of the input parameters in CNT synthesis in controling the CNT structure is highlighted by the recent reports of chirality-control growth [5], and the selective growth of both semiconductor and metal SWCNTs [6,7,8,9].

An additional level of complexity arises from the fact that the output structural features are interdependent. For example, the interdependency between diameter and density has been reported [4]. For the commonly used metal thin film-particle as a catalyst, the diameter and density are inversely correlated where low/high density forests would consist of large/small diameter SWCNTs which we propose as a general rule for the structural control of SWCNT forests. A strong link between density and the degree of alignment has also been revealed, where SWCNTs do not stand vertically because individual SWCNTs lack the rigidity to independently stand, and at large spacing the “crowding effect” needed to laterally confine the SWNTs to form a self-assembled vertically-aligned structure is lacking [10,11,12].

As exemplified by structural parameter control, the CNT synthesis can appear very complex. Therefore, it would be of crucial advantage to understand the fundamental growth mechanisms and limiting processes which would help us to understand the complicated interdependency among structural parameters and would serve as a guide to achieve further control of structural parameters. For example, acetylene, among all the hydrocarbons used for CNT synthesis, was shown to be the primary component in CNT growth [13,14,15,16,17]. In fact, highly efficient forest growth has been achieved using only acetylene, whereas water is required to achieve high yield with other carbon feedstocks [18]. The fundamental mechanism governing the catalyst size has been also extensively studied. Amama *et al.* showed the impact of the gas environment affecting the Ostwald ripening behavior of catalyst particle from metal thin film [19]. Kim *et al.* showed that the subsurface diffusion of catalyst into the substrate strongly affects the catalyst volume, and results in growth termination [20]. We interpret that the offsetting effects of Ostwald ripening and subsurface diffusion resulted in the growth of SWNT forests with similar average diameters [21]. Each of these proposed growth mechanisms and processes have contributed to constructing a general picture of CNT synthesis.

In this letter, we have studied the growth kinetics of a series of SWCNT forests spanning a wide range of the diameters (1.9–3.2 nm), and have revealed an additional fundamental growth limiting process where the mass of the individual SWCNT is determined by the individual catalyst volume.

## 2. Results and Discussion

**Figure 1:**
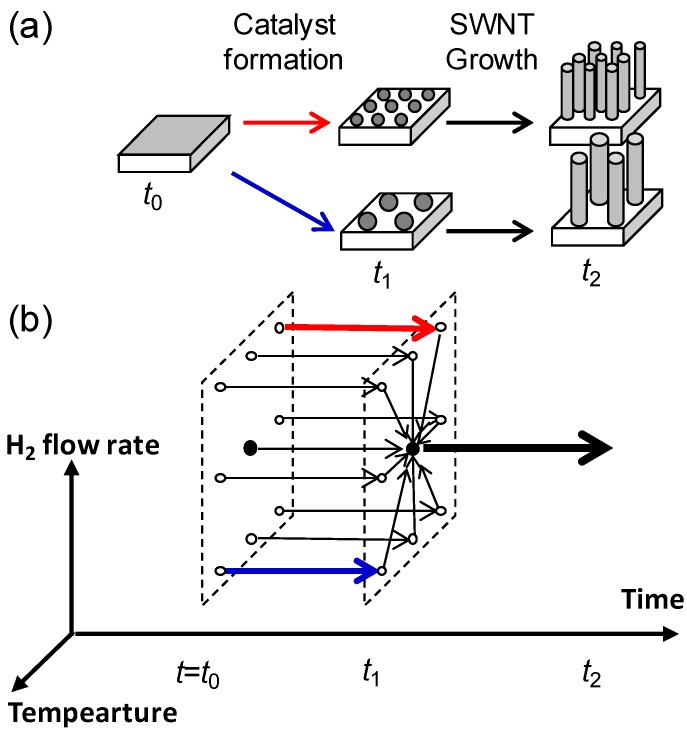
(**a**) Conceptual representation of the process flow of the synthesis of a series of single-walled carbon nanotube (SWCNT) forest with a continuous and wide range of structural properties. Structural control of SWCNT forests was achieved by decoupling the catalyst nanoparticle formation process from the CNT growth process; (**b**) Schimatic representation of the series of conditions for catalyst formation process. Conditions shown by red/blue arrows (large/low H_2_ flow rate and low/high temperature) resulted in high/low density and small/large diameter SWCNT forest.

In this work, we synthesized a series of SWCNT forests with continuous and wide range of the diameter and density and studied the growth kinetics from which we found the fundamental growth limiting process. We achieved structural control of SWCNT forests by decoupling the catalyst nanoparticle formation process from the CNT growth process, as reported in elsewhere [4]. This aspect is conceptually shown in the process flow of the catalyst formation and CNT synthesis processes (Figure 1a). We used an infrared lamp furnace and controlled the process temperature, total gas flow (TF) and H_2_ concentration of the catalyst formation process (Figure 1b). Catalyst substrate was exposed to hydrogen with different flow rates at the target temperature (defined as formation temperature). This process is defined as the catalyst formation process. Highly efficient SWCNT forest growth was carried out immediately after the catalyst formation process at a fixed growth condition. The average diameters of the SWNTs in each forest were evaluated by Fourier transform infrared spectroscopy method with the support of transmission electron microscope (TEM) and atomic force microscope studies, which is described elsewhere [4].

**Figure 2:**
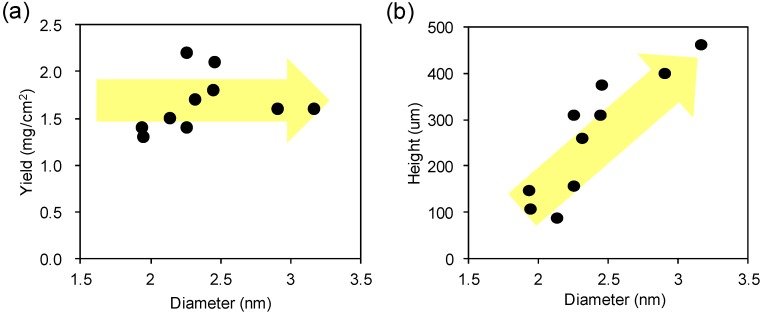

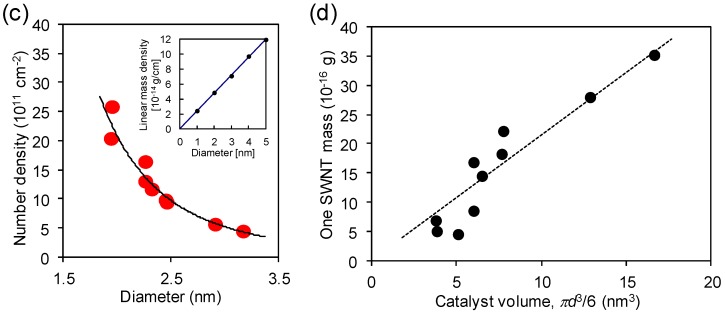
(**a**) Plot of the yield *versus* the diameter of SWCNT; (**b**) Plot of the forest height *versus* the diameter of SWCNT; (**c**) A plot of the catalyst number density as a function of the SWCNT diameter. Dotted line represents an inverse cubic power law: $n=n_{0}{\left(d/d_{0}\right)}^{-3.4}$, where *n*_0_ = 2.2 × 10^13^ cm^−^^2^ and *d*_0_ is 1 nm. Inset shows the Plot of linear mass density as a function of diameter; (**d**) Plot of the individual SWCNT mass* versus* the catalyst volume. Dotted line represents a linear relationship with a slope of 2 × 10^−^^16^ g/nm^3^.

By this method, we could control the diameter of the catalyst nanoparticles and thus grow SWCNT forests with different diameter and density. One fundamental interdependency was observed between the diameter and density, where small diameter SWCNTs possessed high density forests while large diameter SWCNTs possessed low density forests, as shown below (Figure 2c). In this paper, to concentrate on the interdependency between structural parameters, we do not provide the specific experimental conditions used to achieve these data points. Briefly, the small diameter/high density SWCNT forests were grown by using low formation temperature and large hydrogen flow, and the large diameter/low density SWCNT forests were grown by high formation temperature and small hydrogen flow. A plot of the catalyst number density as a function of the SWCNT diameter exhibited an inverse cubic relationship.

**Figure 3:**
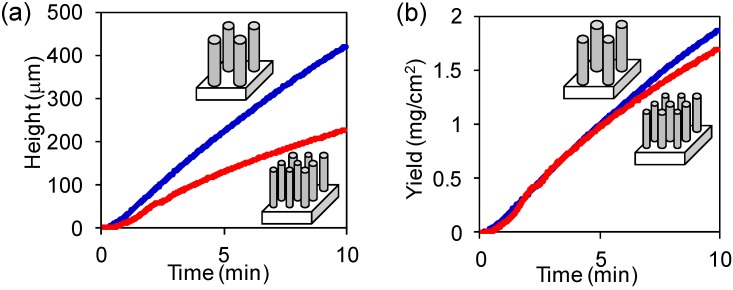
(**a**) The evolutions of two forest heights as a function of time measured by in-situ height monitor using a commercially available telecentric optical system. Blue and red lines represent small diameter (1.9 nm) and large diameter (2.6 nm) SWCNT forest, respectively; (**b**) The evolutions of forest yield, which was obtained by multiplying the heights with the densities.

We chose two growth conditions representing the extreme ends of the structural properties (diameter: 1.9 nm, number density: 2.0 × 10^12^ cm^−^^2^, and diameter: 2.6 nm, number density: 6.0 × 10^11^ cm^−^^2^) and measured the *in-situ* growth height evolution and plotted the height as a function of time by using a commercially available telecentric optical height monitoring system (LS7030M, Keyence, Osaka, Japan) [22]. Throughout the growth span, the high density forest exhibited a slower growth rate when compared to the low density forest (Figure 3a). The heights of forests were converted to yields (CNT weight per substrate area) by multiplying the heights with the CNT forest densities, which were measured *ex-situ* after growth (Figure 3b). Previous research has addressed that the density remains roughly constant throughout the structure of the SWCNT forest which means that at each growth time, the density of the SWCNT forest is fairly constant [23]. This is because the density is determined mainly by the catalyst density and catalyst activity which does not change much within this growth span.

Central to this work, we found that the yield evolution of the two forests converged onto the same line. This demonstrated that although these two forest structures were different in both diameter and density, *etc*., and the actual production rate of SWCNTs was constant. Further, we note that these two forests, which possess different structures, started from the same catalyst thin film and differed only by the catalyst formation processes. Previous analysis showed that the total catalyst volume was constant for these two cases [4]. From this we can conclude that for these two different types of SWCNT forests, the SWCNT production yield per Fe catalyst volume was constant. From an atomistic standpoint, the amount of CNTs produced from each Fe atom was constant. This implies that the conversion ratio of carbon sources by Fe catalyst was the rate-limiting process for these growths. This fundamental knowledge guides us as to what can practically be achieved. In short, the conversion rate of carbon feedstock into CNTs was constant for these two extreme cases.

We extended our investigation by applying this characterization to a series of SWCNT forests with different average diameters spanning from 1.9 to 3.2 nm, and measured the growth yields with a growth time of 10 min. A plot of the yield* versus* the diameter of SWCNT shows that the yield was relatively constant (~1.5 mg/cm^2^) across the diameter range (Figure 2a). Next, we plotted the height* versus* diameter showing a monotonic relationship in Figure 2b (note that this relationship is not linear). Third, the number density was estimated by dividing the yield by the average mass of individual SWCNT, which was calculated by multiplying forest height and the linear mass density (CNT mass per length) of the SWCNTs. We note that the observed yields are assumed to originate from SWCNT, based on TEM studies [4]. As previously reported, linear mass density increases linearly with diameter (inset of Figure 3c), because larger CNTs possess larger circumference and therefore more carbon atoms. The number density plotted as a function of diameter shows the density of CNTs decreases as an inverse cubic with increased diameter (Figure 2c).

From these parameters which describe the structural parameters of the CNT forest ensemble, we focus our discussion to a single CNT from a single catalyst. In doing so, we assume that the CNT diameter corresponds to the catalyst diameter, as experimentally demonstrated in previous reports [1]. Second we assume 100% catalyst activity, which is close to the previously experimentally observed 84% [23]. We also estimated the catalyst volume as π*d*^3^/6, assuming that the catalyst is a sphere with diameter, *d*. Importantly, a linear relationship was observed when plotting the individual SWCNT mass* versus* the catalyst volume (Figure 2d). This result showed complete agreement with the in-situ height monitoring results (Figure 3b) and means that the SWCNT production rate is a linear function of the number of Fe atoms in an individual catalyst. In other words, the conversion ratio per Fe atom, whether incorporated in a large or small catalyst, is constant and therefore we propose that it represents the rate-limiting process for SWCNT forest growth. We calculated the slope in Figure 2d as 2 × 10^−^^16^ g/nm^3^, which means each Fe atoms is converting 2 × 10^2^ carbon amount into CNTs per one second. We think that our result suggest that the bulk diffusion of carbon is the origin of the rate-limiting process, at least our current experimental conditions. We also believe that the value of this slope would be critically changed by the input growth parameters, because the previously reported value (3.5 × 10^21^ C atoms/s g Fe) is only 0.1% of our value [24]. This rate limiting process implies that the mass of the individual SWCNT is determined by the individual catalyst volume, and consequently the forest yield is determined by the total catalyst volume, not by how the volume is distributed. It also implies that the larger diameter SWCNTs will provide larger length which is more suited for mass production. More specifically, the growth rate of SWCNT with the diameter of 3 nm would be about 10 times faster (40 μm/min, estimated by using the above calculated conversion rate) than SWCNT with the diameter of 1 nm (4 μm/min).

## 3. Experimental Section

As a well-established catalyst for SWCNT forest growth, Fe thin film (1.5 nm) supported on an AlO*_x_* layer (30 nm) sequentially sputtered on SiO_2_/Si substrate was used. This substrate was exposed to hydrogen (90%, diluted with He) with different flow rates (500−4000 sccm) at the target temperature (500−950 °C, defined as formation temperature). This process is defined as the catalyst formation process. Water-assisted chemical vapor deposition was carried immediately after the catalyst formation process at a fixed growth condition (50 sccm C_2_H_4_, 950 sccm He, and ~100 ppm H_2_O for 10 min at 840 °C), well established for highly efficient SWCNT forest growth. The average diameters of the SWNT forests were evaluated by Fourier transform infrared spectroscopy [4].

## 4. Conclusions

In this letter, we have studied the growth kinetics of SWCNT forests from catalyst arrays with various average size and particle number density. We have also revealed an additional fundamental growth limiting process where the mass of the individual SWCNT is linearly increased by the individual catalyst volume, although other structural factors of the catalyst might also affect the catalyst properties. This rate limiting process has several implications regarding fundamental interdependency among the structural character of a SWCNT forest. It would nicely explain the empirical difficulty in growing very tall SWCNT forests with small diameter (~1 nm) while most of the long SWCNT forest were grown from larger diameter (~3 nm) SWCNTs. It also implies that, for forests with similar number density, the larger diameter CNTs will provide larger length and yield which is more suited for mass production.
